# Anisotropic optical responses of layered thallium arsenic sulfosalt gillulyite

**DOI:** 10.1038/s41598-021-01542-6

**Published:** 2021-11-09

**Authors:** Ravi P. N. Tripathi, Jie Gao, Xiaodong Yang

**Affiliations:** 1 Department of Mechanical and Aerospace Engineering, Missouri University of Science and Technology, Rolla, MO 65409 USA; 2 Department of Mechanical Engineering, Stony Brook University, Stony Brook, NY 11794 USA

**Keywords:** Two-dimensional materials, Integrated optics

## Abstract

Multi-element two-dimensional (2D) materials hold great promise in the context of tailoring the physical and chemical properties of the materials via stoichiometric engineering. However, the rational and controllable synthesis of complex 2D materials remains a challenge. Herein, we demonstrate the preparation of large-area thin quaternary 2D material flakes via mechanical exfoliation from a naturally occurring bulk crystal named gillulyite. Furthermore, the anisotropic linear and nonlinear optical properties including anisotropic Raman scattering, linear dichroism, and anisotropic third-harmonic generation (THG) of the exfoliated gillulyite flakes are investigated. The observed highly anisotropic optical properties originate from the reduced in-plane crystal symmetry. Additionally, the third-order nonlinear susceptibility of gillulyite crystal is retrieved from the measured thickness-dependent THG emission. We anticipate that the demonstrated strong anisotropic linear and nonlinear optical responses of gillulyite crystal will facilitate the better understanding of light-matter interaction in quaternary 2D materials and its implications in technological innovations such as photodetectors, frequency modulators, nonlinear optical signal processors, and solar cell applications.

## Introduction

Two-dimensional (2D) materials have paved an effective way for driving nanophotonics beyond the subwavelength scale^1,2^. This naturally stems from their unusual chemical and physical properties such as high surface-bulk ratios, dangling-bond-free smooth surface^1^, interlayer coupling, and unique electronic and optical responses^3^. Since the successful demonstration of creating stable monolayer and few-layers van der Waals materials in 2004^4^, substantial efforts have been dedicated in exploring their prospects in advancing technological innovations ranging from energy storage to electronic and optoelectronic devices^5–8^. Nevertheless, the collection of the explored 2D materials is primarily confined to mono- and binary-element materials^9–11^ such as black phosphorous, bismuth, tellurium, antimony, GeS, GeAs, ReS_2_, and transition metal dichalcogenides. However, to meet the requirement of future electronic and optoelectronic technologies, it is imperative to expand the landscape of the existing library of mono- and binary-element materials to multi-element 2D materials^9,12^. Moreover, the induction of novel complex nanomaterials would also allow the increased adaptability and versatility of the 2D materials world. In recent years, multi-element 2D materials^5,9,12,13^ with their unconventional physical properties such as linear dichroism transition^14^, exceptional charge carrier mobility^15,16^, favorable band structure^17^, and bandgap transition have gained significant attention in the context of ultrafast photonics applications^18,19^. However, research on complex multi-element 2D materials is still in the nascent phase, and the fundamental understanding of their structural and electronic properties and other microscopic processes which affect the linear and nonlinear optical responses in these materials is very limited.

Motivated by this, herein we introduce gillulyite as a new type of sulfosalt-based anisotropic 2D layered materials to further expand the currently existing 2D material library. In general, sulfosalts are complex mixed-metal chalcogenides with the general composition A_m_B_n_C_p_, in which A represents metals such as lead, silver, copper, tin, iron, manganese, mercury or thallium, B is semimetals like arsenic, antimony or bismuth, and C is sulfur or selenium^20,21^. Gillulyite is a thallium arsenic sulfosalt with miner antimony, with the idealized chemical formula of Tl_2_(As,Sb)_8_S_13_, which was found in 1991 at the Mercur gold deposit in Oquirrh Mountains, Tooele County, Utah and named in honor of the geologist James C. Gilluly^22^. Gillulyite occurs primarily as cleavable masses in vuggy barite and has a Mohs scale hardness of 2.0 to 2.5. Gillulyite is translucent and has a deep red to maroon color. It is worth noting that the crystallographic and structural properties of bulk gillulyite crystal have been studied, but the light-matter interaction correlated with the structural parameters of the crystal are still unknown. In general, thallium containing compounds are recently identified as potential materials for several important applications ranging from energy storage to security purposes such as thermoelectrics, laser frequency modulators, radiation sensors, and photovoltaics^23–25^. Therefore, the insightful understanding of light-matter interaction associated with the structural properties of these materials will be valuable for their engagement in technological improvisation. To address these issues, here we explore the anisotropic linear and nonlinear optical responses of mechanically exfoliated gillulyite thin flakes. It is noted that although CVD-grown thin films have been developed to a level of centimeter-scale size, the CVD-based growth of large-size thin films of multi-element layered materials with rational and controllable chemical compositions is still an unaddressed issue. As an alternate, naturally occurring multi-element vdW layered minerals pave an interesting way to prepare ultrathin flakes via mechanical exfoliation. However, in general the mechanical exfoliation of these naturally occurring bulk crystals in the large-size range of tens of micrometers is difficult. Here, we demonstrate that it is relatively easy to mechanically exfoliate the naturally occurring bulk gillulyite crystal into large-area thin flakes with sizes of tens of micrometers down to deep-subwavelength scale thicknesses. We further characterize the gillulyite crystal using high-resolution transmission electron microscope (HRTEM) and energy dispersive X-ray spectroscopy (EDXS) techniques to determine the structural and chemical composition information. Subsequently, the anisotropic vibrational and linear optical properties due to the low in-plane lattice symmetry are probed using polarization-resolved Raman and optical absorption spectroscopy. Furthermore, the anisotropic THG response of gillulyite crystal with respect to the incident linear polarization of pump beam is investigated and the third-order nonlinear susceptibility is retrieved from the thickness-dependent THG emission. To obtain further intuitive understanding, the third-order nonlinear susceptibility tensor elements are also estimated by corroborating the measured THG signal with a theoretical nonlinear model. These results will facilitate the fundamental understanding of light-matter interaction in quaternary 2D materials and advance future technological innovations in photonics and optoelectronics such as photodetection, frequency conversion, nonlinear optical signal processing, and photonic circuits.

## Results

### Structural and chemical composition determination of gillulyite crystal

**Figure 1:**
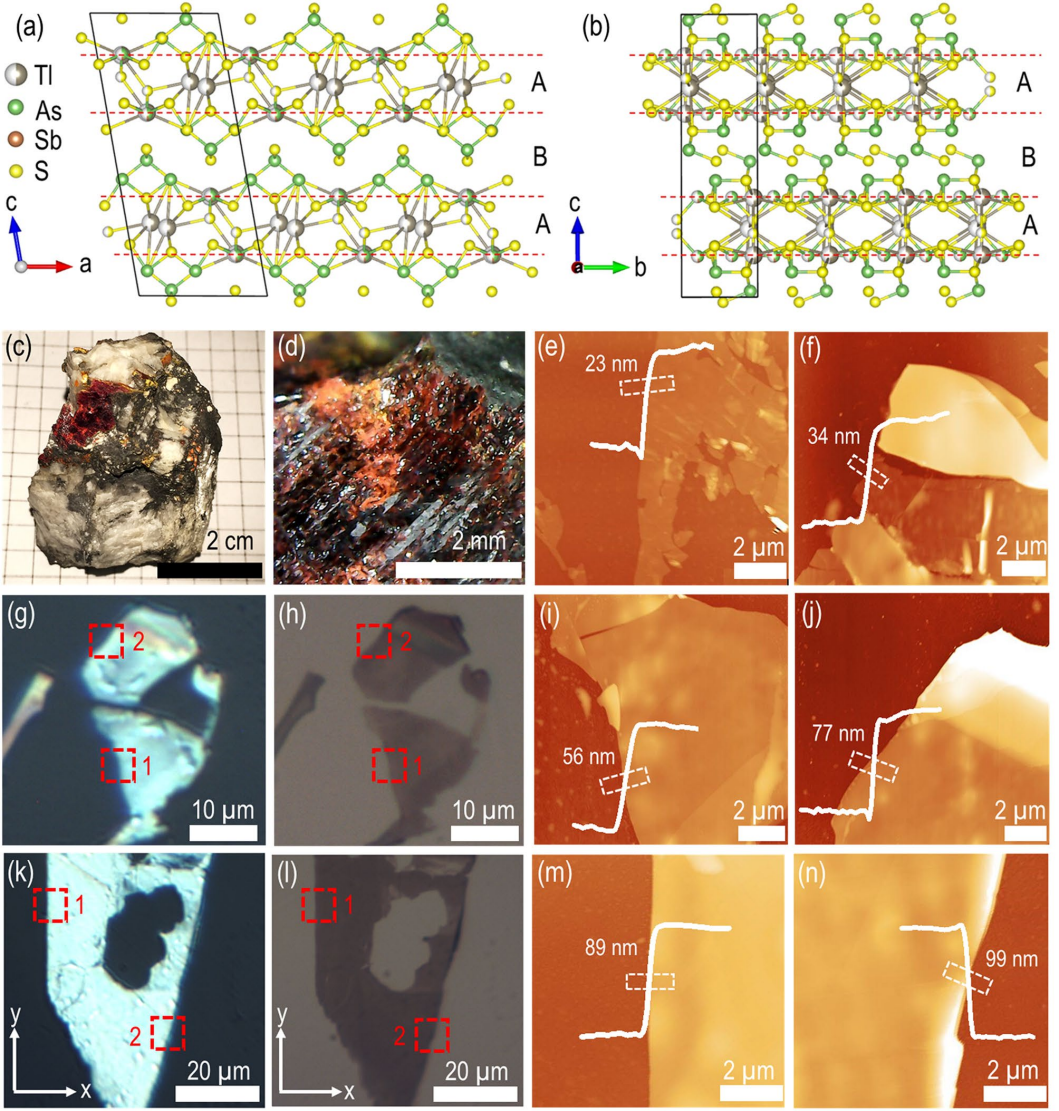
Gillulyite crystal structure and mechanically exfoliated thin flakes. (**a**,**b**) Schematic diagram of gillulyite crystal structure from two side views. (**c**) Image of a typical bulk natural gillulyite mineral. (**d**) Magnified view of the bulk crystal shown in (**c**). (**e**,**f**) AFM images of two exfoliated gillulyite thin flakes of 23 nm and 34 nm. (**g**, **k**) Optical reflection microscopic images and (**h**,**l**) optical transmission microscopic images of mechanically exfoliated large-area thin flakes on glass substrate with different thicknesses of 56 nm, 77 nm, 89 nm and 99 nm. Red dashed-line boxes indicate the scanned region 1 and region 2 for AFM images, whereas *x*-axis and *y*-axis signify the crystal’s axis, respectively. (**i**,**m**) AFM images of the marked region 1 and (**j**,**n**) AFM images of the marked region 2 in the reflection and transmission microscope images. The AFM line profiles signify the thickness and smoothness of the investigated flake region.

Gillulyite belongs to the monoclinic crystal system with space group *P*2/*n* and the unit cell dimensions of *a* = 9.584 Å, *b* = 5.679 Å, *c* = 21.501 Å, *α* = *γ* = 90° and *β* = 100.07°. Figure 1a,b present the side views of the crystal structure of gillulyite projected along the *b*-axis and *a*-axis. The crystal structure of gillulyite can be described by two alternating Tl-containing A layer and PbS-like B layer along the *c*-direction^26–28^. In the Tl-bearing A layer with partly zeolitic character, TlS_5_ and As_2_S_5_ groups alternate regularly along the *b-*direction. The B layer can be described as a substantially distorted PbS-like motif which is periodically twinned by insertion of another (As,Sb) polyhedron, and is filled with lone electron pairs and weak interactions. The order–disorder phenomena happen along the *b*-direction which is caused by ambiguity in the position of TlS_5_-As_2_S_5_ sequences. Complex loop-branched zigzag (As,Sb)_4_S_7_ double-chains of AsS_3_ pyramids are formed along the *a*-direction in the B layer of gillulyite. It is noted that the crystal structure of gillulyite is homeotypic with those of gerstleyite Na_2_(Sb,As)_8_S_13_⋅2H_2_O^29^ and ambrinoite (K,NH_4_)_2_(As,Sb)_8_S_13_·H_2_O^30^. The weak interactions in the B layer of gillulyite will lead to a small interlayer cohesive energy, indicating the suitability for mechanical exfoliation of the bulk mineral.

Figure 1c shows a picture of a gillulyite mineral rock, where an aggregate of vitreous deep-red platy crystals of gillulyite with a large stepped cleaved surface is presented on massive white barite. Further, the magnified view of deep-red platy crystals of gillulyite is displayed in Fig. 1d. We create the gillulyite thin flakes with different thicknesses on glass substrate via the mechanical exfoliation of the bulk natural gillulyite mineral (from Lulu Cut, South Mercur Pit, Mercur District, Oquirrh Mountains, Tooele County, Utah, USA) with Nitto tape (SPV 224). Figure 1e,f show the atomic force microscopy (AFM) images of two prepared gillulyite flakes with the thicknesses of 23 nm and 34 nm, which validate that gillulyite bulk crystal is exfoliable down to few-layer thick flakes. Large-area thin flakes with high aspect ratios can be obtained by cautiously performing the mechanical exfoliation process. Figure 1g,h display the optical reflection and transmission microscope images of two large-area thin flakes, as highlighted in region 1 with lateral dimension of 12 µm and thickness of 56 nm, as well as in region 2 with lateral dimension of 14 µm and thickness of 77 nm, where the flake thickness and surface smoothness are confirmed by the captured AFM images shown in Fig. 1i,j. Figure 1k–n display two more large-area thin flakes with thicknesses of 89 nm and 99 nm, which reaffirm the consistency of higher aspect ratios attained in the exfoliated gillulyite flakes.

**Figure 2:**
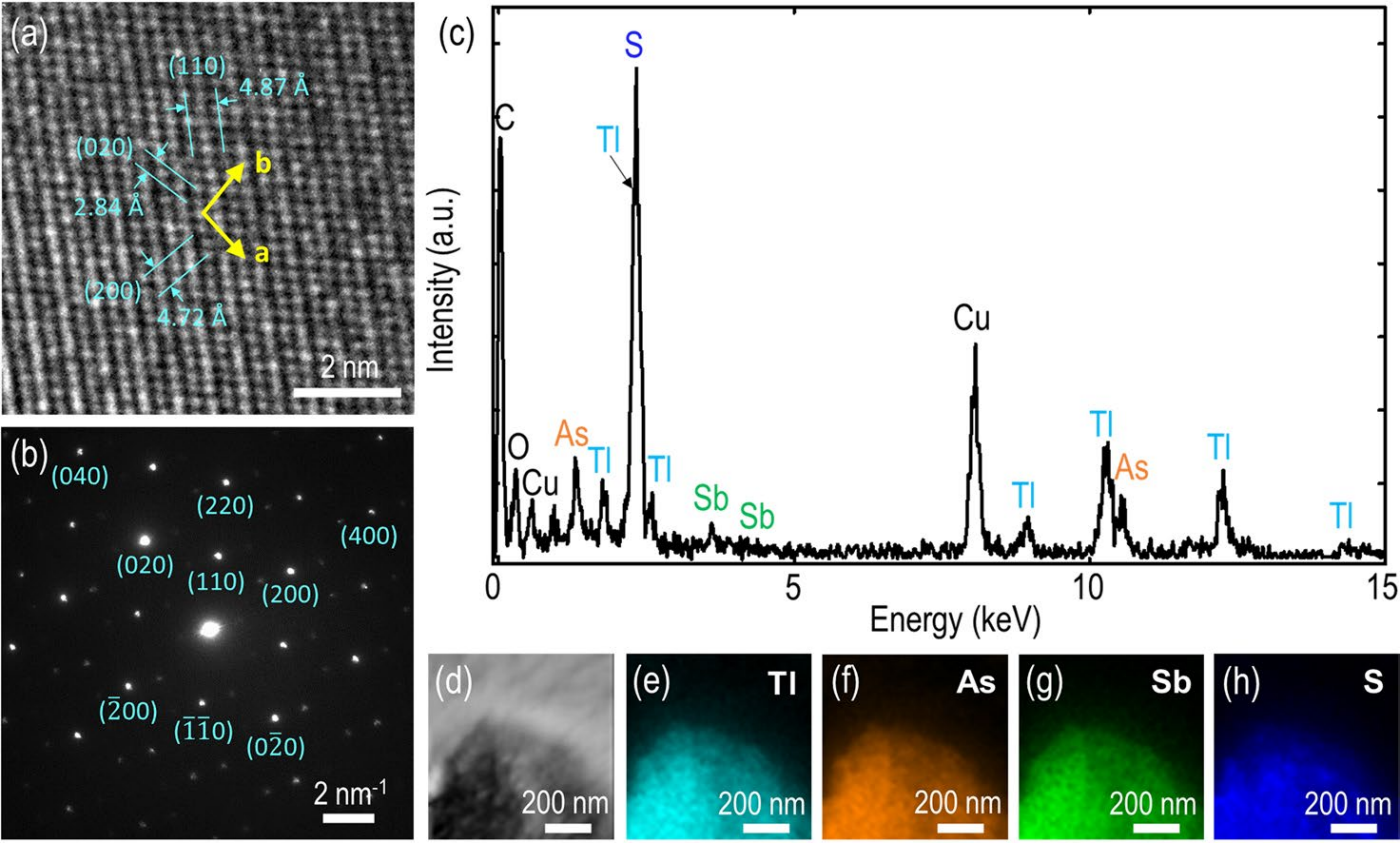
Structural and chemical composition determination of gillulyite crystal. (**a**) Representative atomic HRTEM image of an ultrathin gillulyite flake. (**b**) Corresponding SAED pattern. (**c**) Average EDXS spectrum. The atomic weight percentage of four major elements Tl, As, Sb and S are considered for determining the chemical composition of gillulyite crystal. (**d**) Bright-field TEM image of the probed region. (**e**–**h**) TEM-EDXS mapping of gillulyite crystal showing the presence and uniformity of Tl, As, Sb and S, respectively.

Next, the gillulyite crystal is characterized using HRTEM and EDXS techniques to investigate the structural and chemical composition information. Figure 2a shows a HRTEM image of gillulyite crystal with the determined lattice spacings of ~ 4.72 Å and ~ 2.84 Å and the intersection angle of ~ 97°, which are consistent with the [200] and [020] sets of planes for the monoclinic crystal. The selected area electron diffraction (SAED) pattern in Fig. 2b further confirms the crystalline nature of the exfoliated gillulyite flakes where the spot patterns from the surface normal to the [001] crystal zone axis are displayed. We further quantify the chemical composition of gillulyite crystal. The recorded average EDXS spectrum is shown in Fig. 2c, whereas Fig. 2d–h shows the dark-field TEM image of the scanned region and the corresponding TEM-EDXS elemental maps, emphasizing the homogeneous distribution of thallium (Tl), arsenic (As), antimony (Sb) and sulfur (S) as the prime components in gillulyite crystal. In addition, the presence of copper (Cu) peak in EDX spectrum can be attributed to the TEM grid. The obtained elemental composition (atomic weight %) is summarized in Table 1, which is used to compute the compositional stoichiometry analysis, resulting an empirical formula of Tl_7.89_As_7.80_Sb_1.00_S_13.00_. The accuracy of EDXS estimation method depends upon several important factors such as the nature of probed specimen, the overlap in X-ray emission peaks, and the detection efficiency. Noticeably, the overlap in X-ray emission peaks is a crucial factor under the same operating condition. In our measurements, the X-ray emission peak of thallium is observed at 2.27 keV, whereas the corresponding sulfur peak is detected at 2.31 keV. In the EDXS spectrum, the elemental yield is estimated as per area under the curve for the respective emission peak. Since these two peaks are significantly overlapping with each other, the estimated quantifications of thallium and sulfur with EDXS may not be precise. We anticipate that this is the major reason for the overestimation of thallium abundance and the underestimation of sulfur in the probed specimen. As a result, the empirical formula determined from the EDXS spectrum is not the excellent match with the generic gillulyite chemical formula of Tl_2_(As,Sb)_8_S_13_^22^. Besides, small deviation between these two formulas can be attributed to their geological origins and the presence of carbon (C) and oxygen (O) impurities in naturally occurring minerals. Moreover, such deviation between the empirical and generic formula have been observed in previous studies on naturally occurring 2D materials as well^31,32^.

**Table 1:** EDXS quantification of bulk gillulyite crystal.

Elements	Concentration (at.%)
Tl	26.58 ± 8.57
As	26.27 ± 3.17
Sb	3.38 ± 1.44
S	43.77 ± 4.50

### Polarization-resolved Raman spectroscopy of gillulyite flake

**Figure 3:**
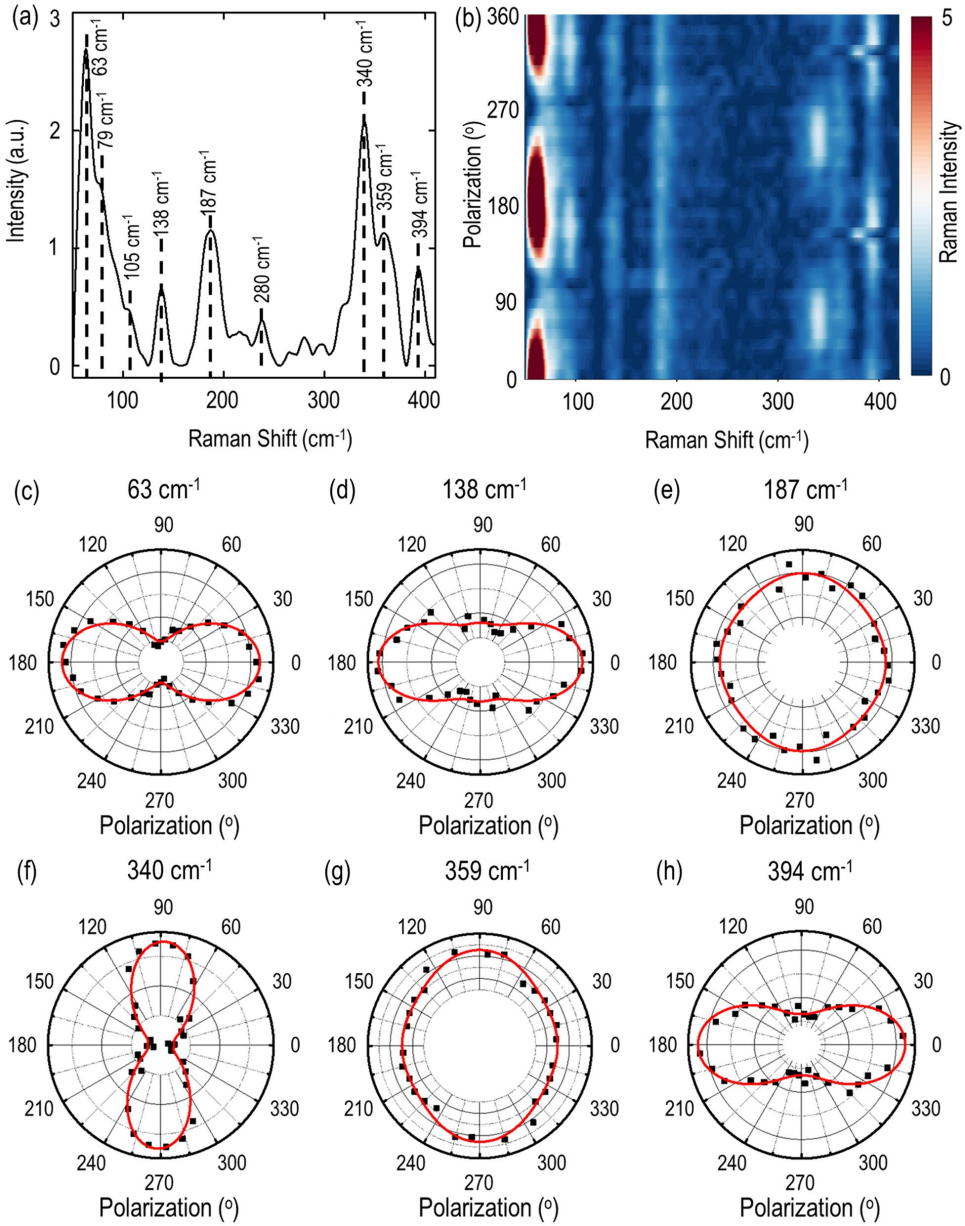
Polarization-resolved Raman characterization of gillulyite flake. (**a**) Recorded Raman spectrum from the 89 nm-thick gillulyite flake. The observed Raman peaks are marked with black dashed lines. (**b**) Color map of polarization-resolved Raman spectra in parallel polarization configuration. (**c**–**h**) Polar plots for different anisotropic Raman modes in parallel polarization configuration at 63, 138, 187, 340, 359 and 394 cm^-1^. Black squares are the experimental data, whereas red solid lines denote the theoretical fits.

In order to understand the vibrational modes in gillulyite flakes, the polarization-resolved Raman spectroscopy is conducted on the 89 nm-thick flake in parallel polarization configuration. Figure 3a shows the recorded Raman spectra with the flake excited using a 632.8 nm He–Ne laser, while the analyzer in the collection path is set to be in the parallel direction with respect to the input linear polarization. The Raman spectrum of gillulyite crystal shows a series of distinct Raman modes within the 50–420 cm^-1^ frequency range, which has evident spectral similarities to the Raman spectra from other thallium arsenic sulfosalts such as lorándite TlAsS_2_, fangite Tl_3_AsS_4_ and rebulite Tl_5_Sb_5_As_8_S_22_, as well as close to the Raman spectrum of orpiment As_2_S_3_, arising from the presence of AsS_3_ trigonal pyramids in the crystal structures^33,34^. The Raman bands between 400 and 220 cm^-1^ include the characteristic four vibrational modes from ν_1_ to ν_4_ of the isolated and interconnected AsS_3_ pyramids with band energies of ν_1_ > ν_3_ > ν_2_ > ν_4_. The modes in the 400–330 cm^-1^ region represent the symmetric and antisymmetric As-S stretching vibrations of ν_1_ and ν_3_, which might overlap to each other or are very close in energy. The modes in the 325–225 cm^-1^ region are the S-As-S bending vibrations of ν_2_ and ν_4_, where the out-of-plane bending vibrations occur above 290 cm^-1^ and the in-plane bending vibrations appear in the low energy range. The Raman bands below 220 cm^-1^ originate from the lattice vibrational modes.

To obtain further insight of anisotropic vibrational modes, the dependence of the Raman modes on the incident linear polarization angle is systematically studied. Gillulyite crystal belongs to the monoclinic crystal family. Taken into considering the crystal symmetry, the Raman tensor for different A_g_ modes can be written as^35^

1 $$R \left( {A_{g} } \right) = \left[ {\begin{array}{*{20}c} {ae^{{i\emptyset_{a} }} } & 0 & {de^{{i\emptyset_{d} }} } \\ 0 & {be^{{i\emptyset_{b} }} } & 0 \\ {de^{{i\emptyset_{d} }} } & 0 & {ce^{{i\emptyset_{c} }} } \\ \end{array} } \right]$$

where *a*, *b*, *c*, and *d* are the amplitudes of Raman tensor elements, and $\emptyset_{a}$, $\emptyset_{b}$, $\emptyset_{c}$, and $\emptyset_{d}$, denote the associated phases. In addition, the Raman modes also show the significant dependence on the unit polarization vectors of the incident and scattered light $e_{i}$ and $e_{s}$, with the Raman intensity of $I \propto { }\left| {e_{i} .R.e_{s} } \right|^{2}$. According to the experimental configuration, $e_{i} = \left( {cos\theta , sin\theta , 0} \right)$ and $e_{s} = \left( {cos\theta , sin\theta , 0} \right)$ for parallel polarization configuration, giving the Raman intensity for A_g_ modes as follows^35^

2 $$I_{{A_{g} }}^{ / /} \propto \left( {\left| a \right|sin^{2} \theta + \left| b \right|\,\,cos\emptyset_{ba} \,\,cos^{2} \theta } \right)^{2} + \left( {\left| b \right|sin\emptyset_{ba} \,cos^{2} \theta } \right)^{2}$$

where // denotes the parallel polarization configuration and $\emptyset_{ba} = \emptyset_{b} - \emptyset_{a}$ denotes the associated phase difference. Figure 3b shows the color map of Raman intensity as per the incident linear polarization angle for the 89 nm-thick flake. It is observed that the Raman modes are anisotropic in nature and the Raman intensity vary periodically as per the incident polarization angle. To obtain further insight on the periodicity of the Raman modes, the experimentally recorded polarization-resolved Raman spectra are theoretically fitted using Eq. (2). Figure 3c–h show the polar plots of the Raman modes at 63, 138, 187, 340, 359 and 394 cm^-1^. The experimental data are denoted as black squares, whereas the theoretical fitting is shown with red solid lines, indicating a good agreement with each other. All the A_g_ modes show anisotropic two-lobe patterns with the Raman intensity maxima either at 0° and 180° or 90° and 270°. Moreover, the orientation of A_g_ modes in parallel polarization configuration indicates the crystal axis of the investigated gillulyite flake^13,36^. Here, the 0° and 90° directions are acknowledged as the *a*-axis (along *x*-axis) and *b*-axis (along *y*-axis) of gillulyite crystal, for further analyzing the anisotropic linear and nonlinear optical properties of the crystal. It is worth noting that the effects of glass substrate on the observed anisotropic Raman modes from gillulyite crystals are negligible (see Supplementary Information).

**Figure 4:**
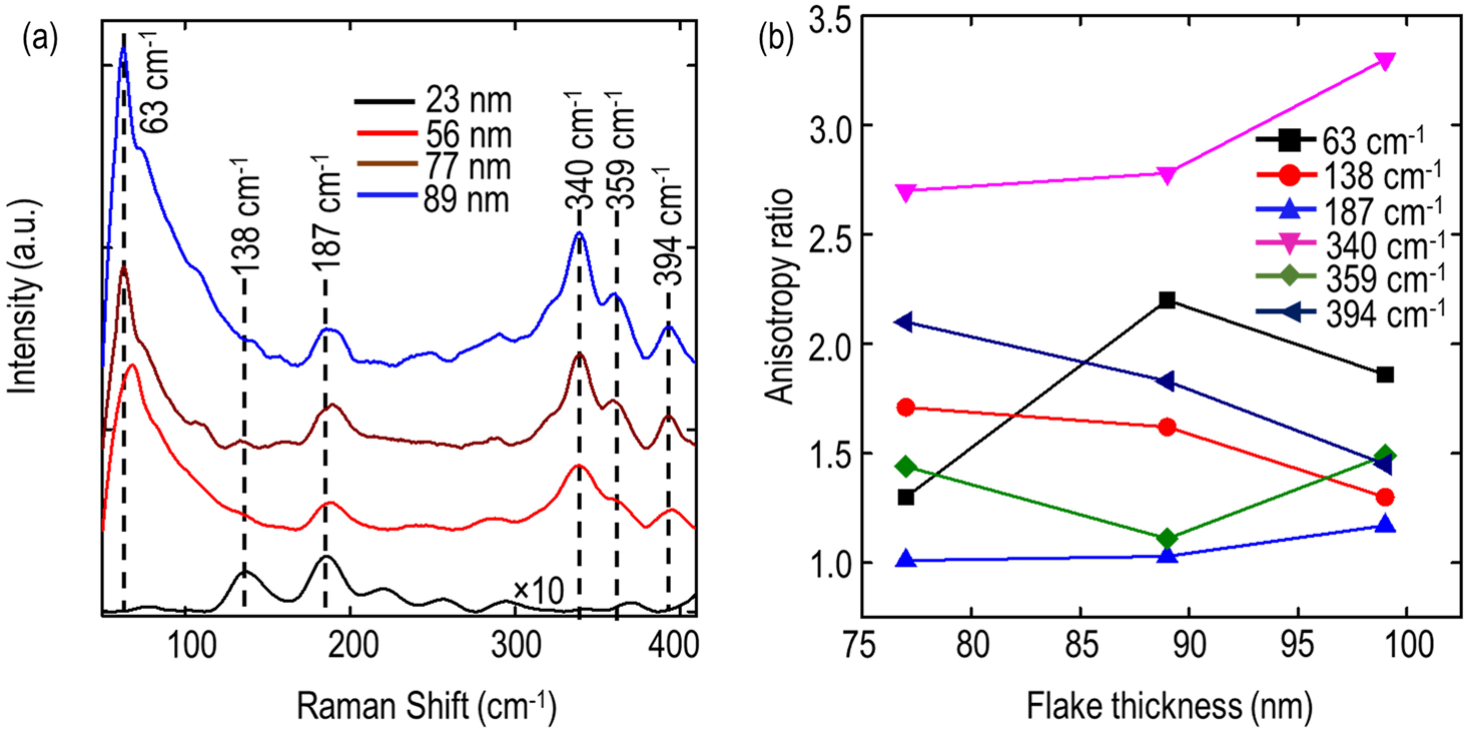
Thickness-dependent Raman characterization of gillulyite flake. (**a**) Measured thickness-dependent Raman spectra of gillulyite crystals. The Raman spectrum of 23 nm gillulyite crystal is multiplied with a factor of 10 to make the comparison visible with other crystals. (**b**) Comparison of anisotropy ratios for Raman A_g_ modes in polarization-resolved Raman spectroscopy.

Furthermore, the Raman spectra of four different gillulyite crystals with thicknesses of 23, 56, 77, and 89 nm are collected under the excitation of linearly-polarized 632.8 nm laser source without the analyzer in the collection path. The collected Raman spectra are shown in Fig. 4a and the Raman A_g_ peaks at 63, 138, 187, 340, 359, and 394 cm^-1^ are indicated with black dashed lines. It is observed that the Raman signal intensity increases with the increased gillulyite crystal thickness. However, there is no significant frequency variation in the observed Raman modes as a function of the crystal thickness. Next, we perform the polarization-dependent Raman spectroscopy of these crystals in parallel polarization configuration and compare the anisotropy ratios of the Raman intensities for the observed A_g_ modes at 63, 138, 187, 340, 359, and 394 cm^-1^. The anisotropy ratio depending on the crystal thickness is plotted in Fig. 4b. It is observed that most of the Raman A_g_ modes are highly anisotropic in nature and the anisotropy ratio just slightly changes as per the probed gillulyite crystal thickness.

### Polarization-resolved optical absorption spectroscopy of gillulyite flake

**Figure 5:**
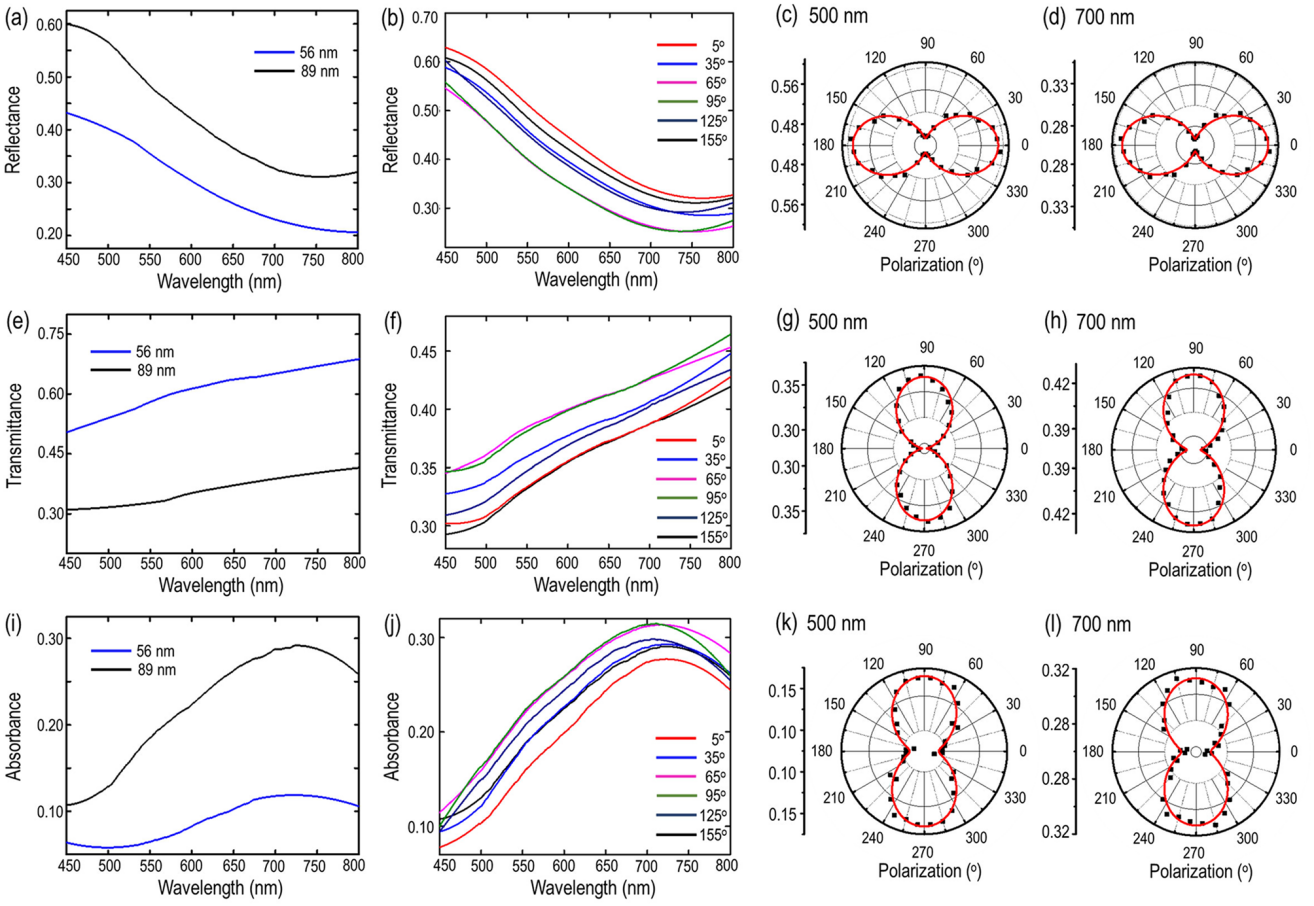
Polarization-resolved optical absorption spectroscopy in gillulyite flakes. (**a**,**e**,**i**) Measured reflectance (*R*), transmittance (*T*), and absorbance (*A*) spectra for two gillulyite flakes with thicknesses of 56 nm (blue solid curves) and 89 nm (black solid curves). (**b**,**f**,**j**) Measured polarization-resolved reflectance, transmittance, and absorbance spectra for the 89 nm-thick gillulyite flake. (**c**,**d**,**g**,**h**,**k**,**l**) Polarization-resolved polar plots of reflectance, transmittance, and absorbance at two different wavelengths of 500 nm and 700 nm. The measured data points are indicted by black squares, while the theoretical fits are plotted with red solid curves.

The prevalent anisotropy in polarization-dependent Raman modes also accredit the presence of linear dichroism in gillulyite crystal. To get further intuition regarding the intrinsic linear dichroism resulted from the low in-plane crystal symmetry, the optical absorption characteristics of two gillulyite flakes with thicknesses of 56 nm and 89 nm are investigated in the visible range from 450 to 800 nm using polarization-resolved optical absorption spectroscopy. Figure 5a,e,i show the measured reflectance (*R*), transmittance (*T*) and absorbance (*A* = 1 − *R* *−* *T*) spectra for these two gillulyite flakes, by keeping the incident linear polarization fixed along *x*-axis of the probed crystal. It is found that the reflectance is higher for the relatively thick flake (89 nm), whereas the transmittance is higher for the thinner flake (56 nm). Interestingly, the reflectance spectra from both flakes show a gradual decrement with a dip around 725 nm, whereas the transmittance increases monotonously within the range of 450 nm to 800 nm. The reflectance dip around 725 nm results in an absorbance peak shown in Fig. 5i, which is related to the optical band gap of gillulyite crystal, giving the mineral its deep red color similar to that of lorándite and fangite^37^. To further investigate the anisotropic features of optical absorption and linear dichroism, polarization-resolved reflectance, transmittance, and absorbance spectra are systematically measured for the 89 nm-thick gillulyite flake, as shown in Fig. 5b,f,j. The linear polarization angle is defined relative to *x*-axis of the crystal. The polar plots of reflectance, transmittance, and absorbance spectra as a function of the linear polarization angle at two different wavelengths of 500 nm and 700 nm are further displayed in Fig. 5c,d,g,h,k,l. The measured data points are theoretically fitted using the formula $\alpha \left(\theta \right)= {\alpha }_{x} {cos}^{2}\left(\theta \right)+ {\alpha }_{y} {sin}^{2}\left(\theta \right)$, where ${\alpha }_{x}$ and ${\alpha }_{y}$ are the magnitudes along *x*-axis and *y*-axis. The obtained reflectance, transmittance, and absorbance anisotropy ratios at 500 nm (700 nm) are *R*_*x*_*/R*_*y*_ = 1.25 (1.35), *T*_*x*_*/T*_*y*_ = 0.82 (0.89), and *A*_*x*_*/A*_*y*_ = 0.65 (0.86), respectively. It is indicated that the gillulyite crystal absorbs photons anisotropically along the directions of *a*-axis (*x*-axis) and *b*-axis (*y*-axis), exhibiting strong wavelength-dependent linear dichroism effects. It is noted that the effects of glass substrate on the reported transmittance, reflectance, absorbance values and the linear dichroism from gillulyite crystals are negligible (see Supplementary Information).

### Anisotropic third-harmonic generation response of gillulyite flake

**Figure 6:**
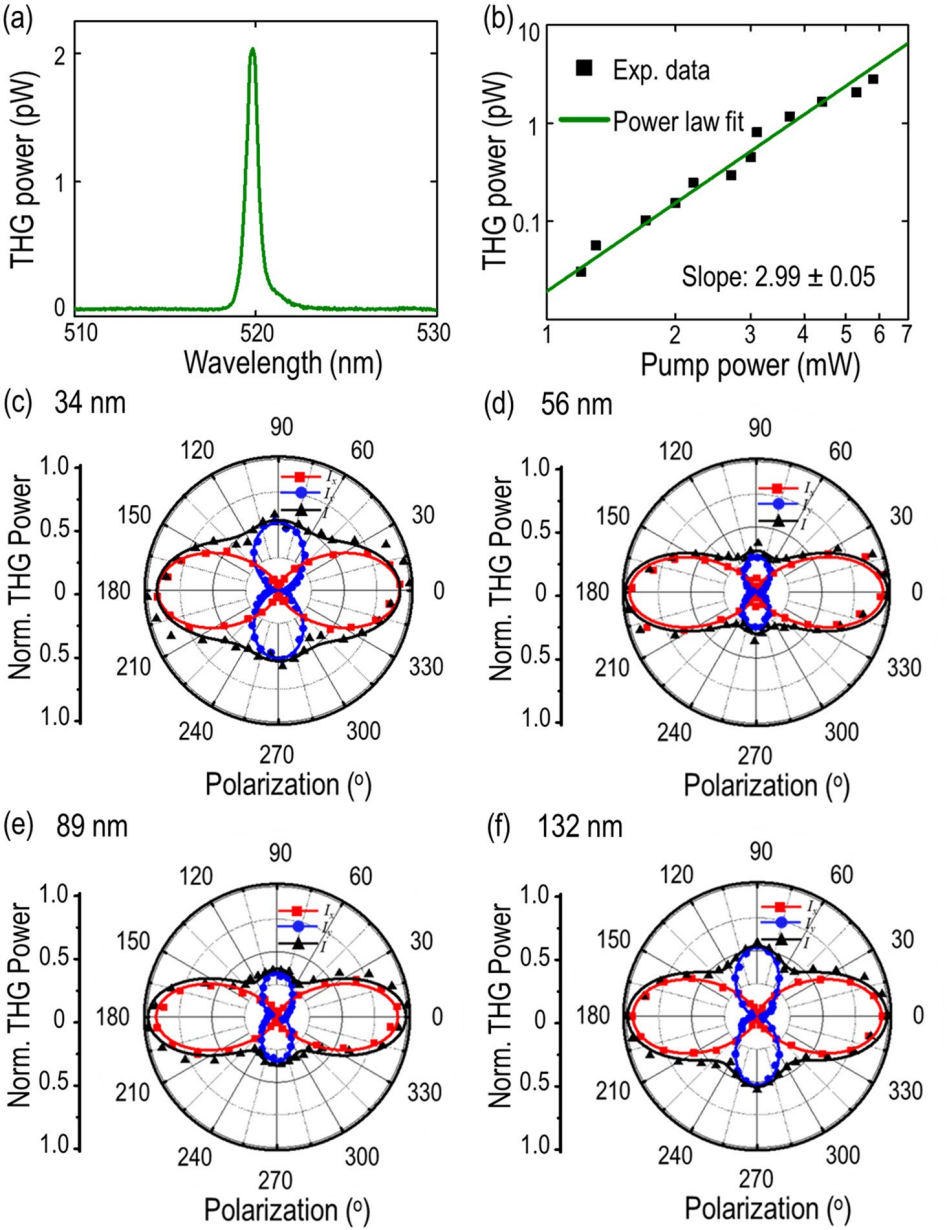
Anisotropic THG emission in gillulyite flakes. (**a**) Recorded THG emission spectrum from the 89 nm-thick gillulyite crystal. (**b**) Logarithmic scale plot of THG emission power as a function of the pump power. Black squares are the measured data points, whereas green solid line represents the cubic power law fit. (**c**–**f**) Polarization-resolved THG emission power for four gillulyite crystals with thicknesses of 34, 56, 89 and 132 nm. Red squares, blue dots and black triangles denote the measured *x*-component ($I_{x}$), *y*-component ($I_{y}$), and total ($I$) THG emission power, respectively. The theoretical fits are plotted as solid curves in the respective colors.

The reduced in-plane symmetry in gillulyite crystal also suggest the high anisotropic nonlinear optical response. The anisotropic THG emission in gillulyite flakes is probed using a 1560 nm pulsed laser with the beam waist of 1.5 µm. Figure 6a shows the measured THG spectrum from the 89 nm-thick gillulyite flake with the emission peaks at 520 nm, which is one-third of the fundamental wavelength. The THG emission process is further confirmed by the cubic power law fit between the THG emission power and the incident pump power, as shown in Fig. 6b. Next, the in-plane anisotropic THG emission as per the incident pump beam polarization is characterized. The desired input polarization is obtained by using a linear polarizer and a rotating half-wave plate in the illumination path. The *x-* and *y-*components of THG emission power are filtered out by introducing an analyzer orientated in parallel (0°) and perpendicular (90°) to the crystal’s *a*-axis.

Figure 6c–f show the measured angular dependence of THG emission power with respect to the incident linear polarization angle for four gillulyite crystals with different thicknesses of 34, 56, 89 and 132 nm. The obtained THG emission patterns are highly anisotropic with four-lobe patterns. The maximum THG emission power is collected at 0° and 180° along the crystal’s *a*-axis (*x*-axis), while the secondary maxima is recorded at 90° and 270° along the crystal’s *b*-axis (*y*-axis). The measured *x*-component, *y*-component and total THG power data points are represented as red squares, blue dots and black upper triangles, respectively, which are further corroborated with the theoretical third-order nonlinear susceptibility model. The third-order nonlinear susceptibility tensor for a monoclinic crystal can be written as follows^38,39^

3 $$\chi^{\left( 3 \right) } = \left[ {\begin{array}{*{20}c} {\chi_{11} } & 0 & {\chi_{13} } \\ 0 & {\chi_{22} } & 0 \\ {\chi_{31} } & 0 & {\chi_{33} } \\ \end{array} \begin{array}{*{20}c} 0 & {\chi_{15} } & {\chi_{16} } \\ {\chi_{24} } & 0 & 0 \\ 0 & {\chi_{35} } & {\chi_{36} } \\ \end{array} \begin{array}{*{20}c} {\chi_{17} } & {\chi_{18} } & 0 \\ 0 & 0 & {\chi_{29} } \\ {\chi_{37} } & {\chi_{38} } & 0 \\ \end{array} \begin{array}{*{20}c} 0 \\ {\chi_{20} } \\ 0 \\ \end{array} } \right]$$

where the first term in subscript 1, 2 and 3 denotes *x*, *y* and *z* respectively and the second subscript refers to the combination of three components as

$$\begin{array}{*{20}c} {xxx} & {yyy} & {zzz} \\ 1 & 2 & 3 \\ \end{array} \begin{array}{*{20}c} {yzz} & {yyz} & {xzz} \\ 4 & 5 & 6 \\ \end{array} \begin{array}{*{20}c} {xxz} & {xyy} & {xxy} \\ 7 & 8 & 9 \\ \end{array} \begin{array}{*{20}c} {xyz} \\ 0 \\ \end{array}$$

Taken the experimental configuration into account, the incident linearly polarized pump beam can be expressed as $\vec{E} = \hat{x}\left( {\left| E \right|cos\theta } \right) + \hat{y}\left( {\left| E \right|sin\theta } \right)$, where $\theta$ is the linear polarization angle relative to the crystal’s *a*-axis. Also, the incident pump beam resides only in the *x–y* plane, therefore the third-order nonlinear susceptibility tensor elements containing *z*-components can be neglected. Hence, the THG intensity components can then be written as ^36,40,41^

4 $$I_{x}^{{\left( {3\omega } \right) }} \propto \left( {\chi_{11} cos^{3} \theta + 3\chi_{18} \,cos\theta \,sin^{2} \theta } \right)^{2}$$

5 $$I_{y}^{{\left( {3\omega } \right) }} \propto \left( {\chi_{22} sin^{3} \theta + 3\chi_{29}\, sin\theta \,cos^{2} \theta } \right)^{2}$$

Equation (4) and (5) are used to fit the measured angular dependence of THG emission. The theoretical fittings are shown as the solid curves in the respective colors in Fig. 6c–f, showing a good agreement with the measured data.

**Figure 7:**
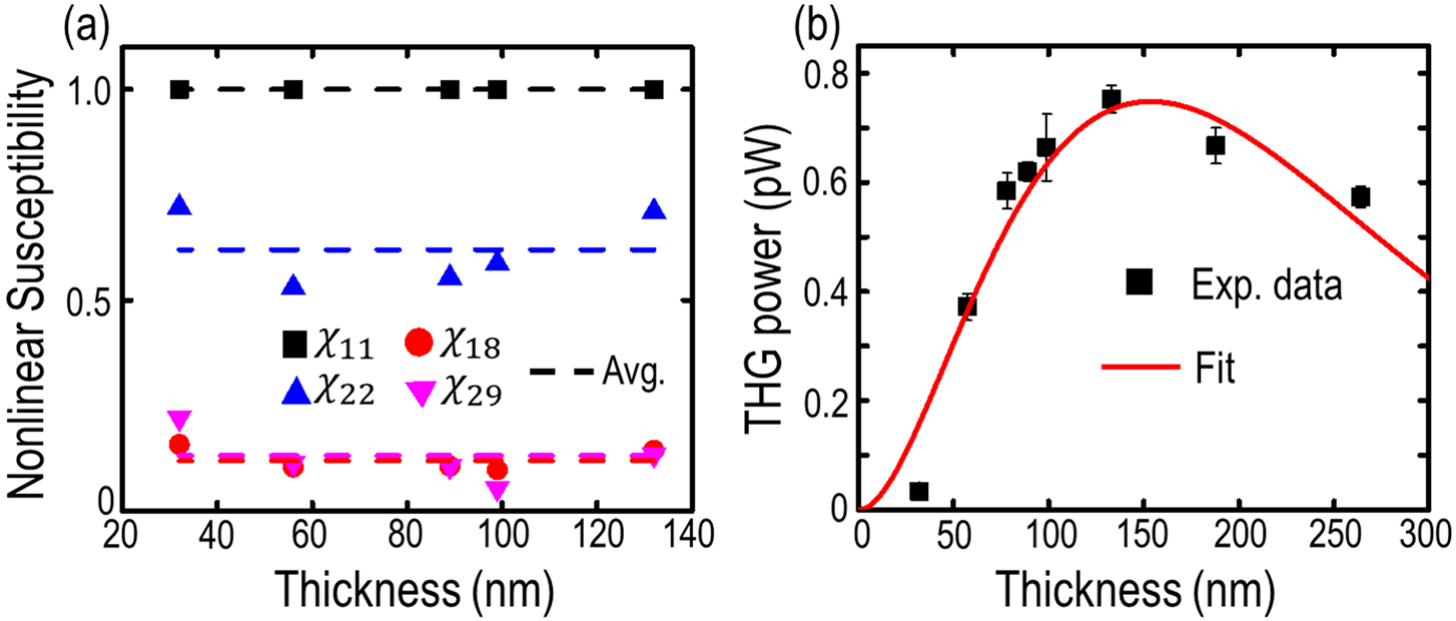
Estimation of third-order nonlinear susceptibility. (**a**) Retrieved relative magnitudes of nonlinear susceptibility tensor elements as a function of the flake thickness, as shown with black squares ($\chi_{11}$), blue upper triangles ($\chi_{22}$), red dots ($\chi_{18}$), and magenta lower triangles ($\chi_{29}$). The average values are represented with dashed lines in the respective colors. (**b**) Thickness-dependent THG emission power. The measured THG emission power is shown with black squares with error bars, whereas the theoretical fitting is plotted as red solid curve.

Additionally, the theoretical fittings enable us to retrieve the relative magnitudes of nonlinear susceptibility tensor elements $\chi_{11}$,$\chi_{18}$,$\chi_{22}$ and $\chi_{29}$, as shown in Fig. 7a for different flake thicknesses. No significant variation in the values of $\chi^{\left( 3 \right) }$ elements is observed for flakes with different thicknesses, and the average values are $\chi_{11} : \chi_{18} :\chi_{22} :\chi_{29} = 1:0.119:0.620:0.131$. Furthermore, the average ratio of $|\chi_{11} |^{2} /|\chi_{22} |^{2}$ indicates the THG anisotropy ratio $I_{x}^{{\left( {3\omega } \right)}} \left( {\theta }= 0 \right)/I_{y }^{{\left( {3\omega } \right)}} \left( {\theta } = 90\right)$ in the gillulyite flakes, which almost remains as a constant of 2.6. Finally, the third-order susceptibility $\chi^{\left( 3 \right) }$ value of gillulyite crystal is estimated. Figure 7b plots the measured THG emission power as a function of the flake thickness. The average power of pump beam is 1.3 mW with 10.22 GW/cm^2^ peak irradiance. The recorded THG emission power gradually increases up to 0.74 pW for the 133 nm-thick gillulyite flake and then decays exponentially. This thickness-dependent THG emission process can be explained in the context of two competitive phenomena of optical gain and loss. In case of the thin flakes, the collected THG emission is proportional to the square of the flake thickness, so that the THG emission power increases for the flake thickness up to around 149 nm. Afterwards, for the thick flakes greater than 149 nm, the optical absorption starts playing a dominant role in attenuating the THG signal propagation, which leads to the exponential decay. The exponentially decayed THG emission further enables us to extract the imaginary part of refractive index (*k*_*3*_) at $\lambda_{3}$ = 520 nm by fitting the thickness-dependent THG emission power equation $P^{{\left( {3\omega } \right)}} \left( l \right) = Ad^{2} \exp \left( { - \frac{{4\pi k_{3} d}}{{\lambda_{3} }}} \right)$ , where *A* is a constant, *d* is the flake thickness. Figure 7b shows the measured data (black squares) and the equation fitting (red curve) with *k*_*3*_ = 0.549. It is worth noting that the real parts of refractive indices of gillulyite correspond closely to those of fangite, in which *n*_*3*_ = 2.81 at 589 nm and *n*_*1*_ = 2.60 at 1553 nm^37,42^. Hence, by considering these refractive index values and other experimental parameters of average pump power $P^{\left( \omega \right)}$ = 1.3 mW, laser pulse width *τ* = 90 fs, repetition rate $f_{rep}$ = 80 MHz, and spot size *W* = 1.5 µm at the fundamental wavelength $\lambda_{1}$ = 1560 nm, the magnitude of $\chi^{\left( 3 \right) }$ can be estimated by the following formula^43^

6 $$P^{{(3\omega )}} (d) = \frac{{9\omega ^{2} d^{2} }}{{16\sqrt {n_{3}^{2} + k_{3}^{2} } n_{1}^{3} \varepsilon _{0}^{2} c^{4} }}\left| {\chi ^{{(3)}} } \right|^{2} \frac{{P^{{(\omega )^{3} }} }}{{f_{{rep}}^{2} W^{4} \tau ^{2} \left[ {\frac{\pi }{{4\ln 2}}} \right]^{3} }}\left( {\frac{{e^{{ - \frac{{4\pi k_{3} d}}{{\lambda_{3} }}}} - 2e^{{ - \frac{{2\pi k_{3} d}}{{\lambda_{3} }}}} + 1}}{{d^{2} \left( {\frac{{4\pi^{2} k_{3}^{2} }}{{\lambda_{3}^{2} }}} \right)}}} \right)e^{{ - \frac{{4\pi k_{3} d}}{{\lambda_{3} }}}}$$

With the real parts of refractive indices of gillulyite crystal $n_{1}$ = 2.60 at $\lambda_{1 }$ = 1560 nm and $n_{3}$ = 2.81 at $\lambda_{3 }$ = 520 nm, the estimated magnitude of $\chi^{\left( 3 \right)}$ for gillulyite crystal is 2.059 × 10^–20^ m^2^/V^2^.

## Discussion

In summary, we have demonstrated the mechanical exfoliation of large-area thin gillulyite flakes of various thicknesses. The structural and chemical composition information of gillulyite crystal has been characterized using HRTEM and EDXS techniques. The anisotropic linear and nonlinear optical properties of gillulyite thin flakes due to the reduced in-plane crystal symmetry have been investigated. The anisotropic Raman modes and linear dichroism have been probed using polarization-resolved Raman and optical absorption spectroscopy. The anisotropic THG emission process in gillulyite crystal has also been explored with the extracted third-order nonlinear susceptibility. In the line of these findings, our presented discussion of gillulyite crystal in the context of anisotropic structural, vibrational, and optical properties will facilitate an insightful comprehension of the light-matter interaction in thallium-contained complex 2D materials as well as pave the way for its implications in photodetection, laser frequency conversion, nonlinear optical signal processing, encrypted optical communication, and photonic circuits.

Finally, the uniqueness in gillulyite crystal structure facilitates an exceptional platform for the wide range tunability of vibrational, optical and electronic properties by comparing with its component binary sulfides via stoichiometric engineering and heteroatoms doping, which will advance many technological innovations. As one thallium-containing sulfosalt compound, gillulyite can be identified as a potential 2D material for realizing several unique applications ranging from thermoelectrics, frequency modulators, to spintronics and radiation sensors. The demonstrated mechanically exfoliated large-area thin gillulyite flakes offer a convenient platform for ultracompact multifunctional flatland optical, electronic and optoelectronic integrated devices, because rational and controllable synthesis of such complex 2D materials remains a challenge till date. Besides, the demonstrated polarization-sensitive vibrational and optical responses in gillulyite crystals indicate that the input polarization can be harnessed as an optical switch in applications such as phonon-based frequency modulation, polarized photodetection, encrypted data transfer, and nonlinear signal processing. With this hindsight, we anticipate that vdW layered sulfosalt mineral gillulyite truly has the potentials to be applied into future integrated photonic, electronic and optoelectronic technologies.

## Methods

### Sample preparation

The sample preparation process involves two steps. In the first step, the glass substrate with 1 cm × 1 cm is treated with deionized water, acetone and isopropanol, followed by ultra-sonication and dried with N_2_ gas. This pretreatment of the glass substrate is repeated several times to ensure the removal of undesired residues on the glass substrate. Further, the glass substrate is heat treated at 150 – 170 °C for 5 min to remove any remnant solvent on the glass surface. In the second step, gillulyite flakes are mechanically exfoliated using Nitto tape (SPV 224) from naturally occurring bulk gillulyite mineral (from Lulu Cut, South Mercur Pit, Mercur District, Oquirrh Mountains, Tooele County, Utah, USA). As the mechanical exfoliation process is a top-down method, it requires multiple efforts to attain the flake thickness of sub-hundred nanometer scale. After completing the exfoliation process, the gillulyite thin flakes are transferred to the pretreated glass substrate followed by heat treatment at 120–130 °C for 2 min. Then, the exfoliated flakes are examined using optical reflection and transmission microscope, and atomic force microscope for estimating the flake thickness. This procedure is repeated multiple times till the desired flake thickness range is obtained. In the view of this, the mechanical exfoliation process can certainly control the thickness range of the prepared flake.

### Polarization-resolved Raman spectroscopy

The sample is illuminated with a 632.8 nm He–Ne laser using a 40 × objective lens (NA = 0.65) and the back-reflected signal is collected to a spectrometer (Horiba, iHR 520) with a beam splitter. The polarization of incident beam is controlled by introducing a linear polarizer and a rotating half-wave plate in the excitation path. The elastic scattered light is filtered out by engaging an edge filter (Semrock, LP02-633RE-25) in the collection path. The collected signal is further passed through a linear polarization analyzer to record the parallel polarization component of the Raman signal.

### Polarization-resolved absorption spectroscopy

A broadband white light source (Thorlabs, SLS201L, 360–2600 nm) is passed through a linear polarizer and focused on the sample with an 80 × objective lens (NA = 0.5). To record the reflection spectra, the back‐reflected light is routed to the spectrometer using a beam splitter, whereas in the case of the transmission spectra, the transmitted light through the sample is collected using another 100 × objective lens (NA = 0.7). The reflection and transmission spectra are normalized with respect to the light source spectrum to obtain the reflectance (*R*) and transmittance (*T*) spectra, so that the absorbance (*A*) spectrum is obtained with *A* = 1 − *R* *−* T.

### Third-harmonic generation measurement

The sample is pumped with a femtosecond laser source at the fundamental wavelength of 1560 nm (repetition rate 80 MHz, pulse width 90 fs) using a 40 × objective lens (NA = 0.65). The transmitted THG signal through the sample is collected by a 100 × objective lens (NA = 0.7). The pump beam is rejected by introducing a shortpass filter in the collection path. The THG signal is then routed to a spectrometer (Horiba, iHR 520) and an imaging camera for recording the spectra and corresponding image.

## Supplementary Information

Supplementary Information.
