# Supplementary material for: Anisotropic optical responses of layered thallium arsenic sulfosalt gillulyite
Source: Sci Rep. 2021 Nov 9;11:22002. doi: 10.1038/s41598-021-01542-6 (PMC8578543; doi:10.1038/s41598-021-01542-6)
Supplement: Supplementary file 1 — Supplementary Information. [file 41598_2021_1542_MOESM1_ESM.pdf]

Supplementary Information

Anisotropic optical responses of layered thallium arsenic sulfosalt gillulyite

Ravi P. N. Tripathi[1], *Jie Gao*[1,2,*] *and Xiaodong Yang*[1,*]

[1]Department of Mechanical and Aerospace Engineering, Missouri University of Science and Technology, Rolla, MO 65409, USA

[2]Department of Mechanical Engineering, Stony Brook University, Stony Brook, NY 11794, USA

*Correspondence and requests for materials should be addressed to: J.G. (email: jie.gao.5@stonybrook.edu) or X.Y. (email: yangxia@mst.edu)

1. Polarization-dependent Raman spectra for glass substrate

Figure 1a shows the recorded Raman spectrum for glass substrate. Noticeably, no significant Raman peak is observed in the recorded spectrum. Next, the polarization-dependent Raman spectra for glass substrate are collected to further elucidate any contribution of glass substrate. Figure 1b and 1c show the recorded polarization-resolved Raman spectra and the corresponding angular plot at 134 cm^{-1} (the selection of this Raman shift value is arbitrary, however the isotropic trend is consistent for all values). No significant Raman intensity variation is observed in the recorded spectra.

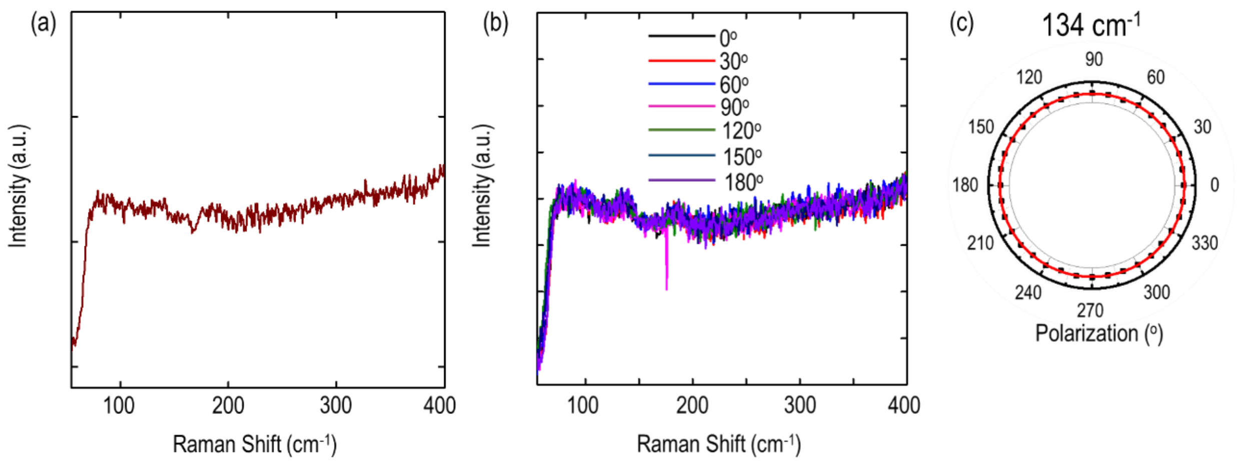

Supplementary Figure 1. (a) Recorded Raman spectrum from glass substrate. (b) Recorded polarization-resolved Raman spectra from glass substrate. (c) Angular plot at 134 cm^{-1} is shown to demonstrate the isotropic nature of Raman response in glass substrate.

2. Polarization-resolved optical absorption spectroscopy for glass substrate

Next, the anisotropic optical properties of engaged glass substrate are measured. Figure 2a shows the recorded transmittance, reflectance, and absorbance spectra in 450 – 800 nm for glass substrate. It is noted that the transmittance is very high (~ 0.87), whereas the reflectance is around ~ 0.06. As a result, the estimated absorbance ranges around ~ 0.07. These values underline the negligible effects of glass substrate on the reported transmittance, reflectance, and absorbance values from gillulyite crystals. To further explore the polarization dependency of glass substrate, the polarization-resolved absorbance measurement is conducted. Figure 2b and 2c show the recorded absorbance spectra and the angular plot at 600 nm (the selection of this wavelength value is arbitrary, however the isotropic trend is consistent for all values). Importantly, the negligible angular variation in absorbance for glass substrate is observed.

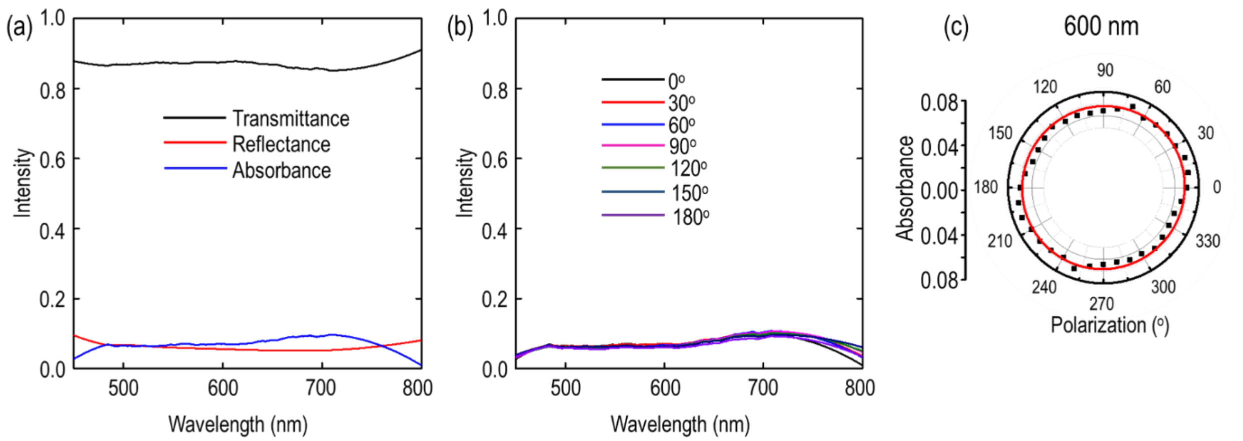

Supplementary Figure 2. (a) Recorded transmittance, reflectance, and absorbance spectra from glass substrate. (b) Recorded polarization-resolved absorbance spectra from glass substrate. (c) Angular plot of absorbance at 600 nm is shown to demonstrate the isotropic nature of absorbance in glass substrate.
